# A cost of illness study of COVID-19 patients and retrospective modelling of potential cost savings when administering remdesivir during the pandemic “first wave” in a German tertiary care hospital

**DOI:** 10.1007/s15010-021-01685-8

**Published:** 2021-08-18

**Authors:** Julia Jeck, Florian Jakobs, Anna Kron, Jennifer Franz, Oliver A. Cornely, Florian Kron

**Affiliations:** 1 VITIS Healthcare Group, Cologne, Germany; 2 Department I of Internal Medicine, Faculty of Medicine and University Hospital Cologne, University of Cologne, Cologne, Germany; 3 National Network Genomic Medicine Lung Cancer, University Hospital Cologne, Cologne, Germany; 4 Faculty of Medicine and University Hospital Cologne, Center for Integrated Oncology (CIO ABCD), University of Cologne, Cologne, Germany; 5 Faculty of Medicine and University Hospital Cologne, Clinical Trials Centre Cologne (ZKS Köln), University of Cologne, Cologne, Germany; 6 Faculty of Medicine and University Hospital Cologne, Chair Translational Research, Cologne Excellence Cluster on Cellular Stress Responses in Aging-Associated Diseases (CECAD), University of Cologne, Cologne, Germany; 7 Faculty of Medicine and University Hospital Cologne, Excellence Center for Medical Mycology (ECMM), University of Cologne, Cologne, Germany; 8 FOM University of Applied Sciences, Essen, Germany; 9 FOM University of Applied Sciences, Aggripinawerft 4, 50678 Cologne, Germany

**Keywords:** COVID-19, SARS-CoV-2, Treatment costs, DRG, Remdesivir, Real-life data

## Abstract

**Purpose:**

First detected in China in 2019, the novel coronavirus disease (COVID-19) has rapidly spread globally. Since then, healthcare systems are exposed to major challenges due to scarce personnel and financial resources. Therefore, this analysis intended to examine treatment costs of COVID-19 inpatients in a German single centre during the first pandemic wave in 2020 from a healthcare payer perspective. Potential cost savings were assessed considering the administration of remdesivir according to the European Medicines Agency label.

**Methods:**

A retrospective medical-chart review was conducted on COVID-19 patients treated at University Hospital Cologne, Germany. Patients were clustered according to an eight-category ordinal scale reflecting different levels of supplemental oxygen. Potential cost savings due to the administration of remdesivir were retrospectively modelled based on a reduced length of stay, as shown in the Adaptive COVID-19 Treatment Trial.

**Results:**

105 COVID-19 patients were identified. There was wide variability in the service data with median treatment costs from EUR 900 to EUR 53,000 per patient, depending on major diagnosis categories and clinical severity. No supplemental oxygen was needed in 40 patients (38.1%). Forty-three (41.0%) patients were treated in intensive-care units, and 30 (69.8%) received invasive ventilation. In our model, in-label administration of remdesivir would have resulted in costs savings of EUR 2100 per COVID-19 inpatient (excluding acquisition costs).

**Conclusion:**

We found that COVID-19 inpatients suffer from heterogeneous disease patterns with a variety of incurred G-DRG tariffs and treatment costs. Theoretically shown in the model, financial resources can be saved by the administration of remdesivir in eligible inpatients.

## Introduction

Since its initial detection in the end of 2019, the novel coronavirus disease (COVID-19) has rapidly spread and is challenging countries, residents, and healthcare systems worldwide [1]. In Germany, Severe Acute Respiratory Syndrome Corona Virus type 2 (SARS-CoV-2) first emerged in late January 2020 [2]. Until now, only a few treatment options have shown positive effects on treating COVID-19 disease. Among antiviral treatments, currently no medication is fully approved and available for use in clinical routine; however, remdesivir was granted a conditional marketing authorisation by the European Medicines Agency (EMA) in July 2020 [3]. Moreover, it is the only antiviral not advised against in the German S3 guideline.^1^ At the same time, however, remdesivir is not recommended for instance by the World Health Organisation [4–6]. As immunomodulatory treatment approaches are considered as especially relevant for advanced, hyper-inflammatory states of disease, the administration of dexamethasone is suggested in severe cases of COVID-19 with oxygen need [4]. At the beginning of 2021, no further antiviral or immunomodulatory treatment methods are recommended outside of clinical trials in Germany.

The German federal government took multiple actions to handle the COVID-19 pandemic and especially to not overburden the healthcare system [7]. This was relevant, because healthcare resources, such as skilled medical staff and intensive-care capacities, were already scarce before the outbreak of COVID-19. Governmental actions included postponing or cancelling elective cases and increasing the number of intensive-care beds equipped for ventilation. According to the German Federal Audit Office, the aforementioned financial subsidies summed up to approximately 10.8 billion Euro in an underlying time period of 1 year which shows the enormous economic burden imposed by the COVID-19 pandemic [8]. Both actions were funded by the federal government with the intentions to disburden healthcare providers and to create spare capacities for the treatment of high-intensive COVID-19 cases [9, 10].

Taking an economic point of view, this analysis intends to help managing scarce financial healthcare resources during the COVID-19 pandemic. To do so, the objective of this study is twofold: first, to evaluate treatment costs of a tertiary care hospital incurred by COVID-19 patients during the first wave of the pandemic from a healthcare payer perspective; second, to assess the economic impact of administering remdesivir according to label.

## Patients and methods

We analysed medical-chart data of real-life COVID-19 inpatient treatment in the University Hospital Cologne from January 01, 2020 to September 30, 2020—herein defined as the first wave of the COVID-19 pandemic in Germany. The analysis was conducted from a healthcare payer perspective, including best-available treatment cost data. Treatment costs for healthcare payer were assumed to correspond the case reimbursement for hospitals according to G-DRG tariffs. As cost data were retrieved from the data warehouse of the University Hospital Cologne, transfer costs, i.e., sick pay, were not available and could thus not be evaluated [11, 12].

### Selection of patients

To identify COVID-19 positive patients, we set specific data extraction parameters. As main inclusion criteria, we used the International Statistical Classification of Diseases (ICD)-10 code for COVID-19 disease U07.1!. The U07.1! code was established by the German Institute for Medical Documentation and Information (DIMDI) in 2020 as a so-called “secondary code” applicable for side diagnoses, which cannot be used alone but needs to be further specified through a main diagnosis [13]. The initial review in the controlling management system was complemented by a case-by-case data review in the hospital information system for all treatment-related clinical information. After completing the patient selection performed by the authors JJ and FK, the process has been individually repeated by another author (JF) to increase data validity and completeness. Participants of the Adaptive COVID-19 Treatment Trial (ACTT-1) (NCT Nr. 04280705)^8^ were excluded, as they might have received remdesivir within the context of the clinical trial. Patients without a valid German health insurance were excluded by the time of analysis to keep the cost data comparable.

Descriptive statistics were used to present demographic variables of patients such as gender and age. Moreover, number of subjects (absolute and relative amount), arithmetic mean, standard deviation, median, and the range indicating minimum and maximum values of a subject were determined using Microsoft Excel (MS 365 Business Standard).

### Characterisation of patients

Two events were defined for patient characterisation and evaluation of the COVID-19 disease course: time of hospitalisation (baseline) and time of maximum disease severity (outcome). In accordance with the eight-category ordinal scale used in the ACTT-1, categories were described as: “Category 1–3”—not hospitalised or hospitalised not requiring supplemental oxygen and no longer requiring ongoing medical care; “Category 4”—hospitalised, not requiring supplemental oxygen but requiring ongoing medical care (related to COVID-19 or to other medical conditions); “Category 5”—hospitalised, requiring any supplemental oxygen; “Category 6”—hospitalised, requiring non-invasive ventilation or use of high-flow oxygen devices; “Category 7”—hospitalised, receiving invasive mechanical ventilation or extracorporeal membrane oxygenation (ECMO); “Category 8”—death [14].

At baseline, all patients were categorised according to the eight-category ordinal scale applied in the ACTT-1, including category 4 to category 7 which are relevant for inpatient treatment. As categories 1–3 cover the outpatient treatment setting or hospitalised patients not requiring COVID-19-related treatment, they reflected exclusive outcome categories in this study. Similarly, category 8 is merely an outcome category and further does not provide insight into the incurred resource consumption in the hospital as it consists of deceased patients [14]. Deceased patients were thus assigned to the outcome categories (4–7) which reflected their maximum disease severity based on their level of requiring supplemental oxygen during their hospital stay. To show the development of disease severity, the distribution of eventually deceased patients across baseline categories was evaluated.

### G-DRG and cost analysis

German diagnosis-related groups (G-DRG) tariff data, herein assumed as treatment costs, are composed of three different cost types: staff costs, material costs, and infrastructure costs. Hospital’s overhead costs are thereby partially included in the infrastructure costs. Cost types are further differentiated by multiple cost centres [15]. Costs are proportionally factored and differ across tariffs. As no direct medical costs were available, G-DRG tariffs of the University Hospital Cologne were used as best-available proxy to allow a systematic evaluation. In this analysis, G-DRG code, length of hospital stay (LOS), case-mix index (CMI), major diagnosis category (MDC), and operation and procedure (OPS) codes were used as underlying parameters for this analysis. Total treatment costs per inpatient case were based on the federal base-rate of North Rhine-Westphalia (2020) of EUR 3654.19 [16]; potential discounts were not included; all cost data are expressed in Euro (EUR) year 2020 values as currency.

### Impact of remdesivir on treatment costs: retrospective model

In the second part of the study, the impact of remdesivir on hospital resource consumption was used to assess the impact on costs. Thereby, we retrospectively modelled its administration during the first wave of the COVID-19 pandemic in Germany according to the label. The label for the use of remdesivir includes adults and adolescents (age ≥ 12 years and weight ≥ 40 kg) with pneumonia requiring supplemental oxygen (low- or high-flow oxygen or other non-invasive ventilation at the start of treatment) [3]. Transferred to the applied eight-category ordinal scale, this corresponded to baseline categories 5 and 6. To evaluate cost savings, we calculated the impact of the reduction of LOS due to remdesivir administration as shown in the ACTT-1 for patients of category 5 (reduction of 2 LOS days) and category 6 (reduction of 4.5 LOS days) [14]. Thereby, acquisition costs of remdesivir were not included in the analysis as healthcare providers were supplied with remdesivir free of charge due to the temporary exemption clause incorporated in the Medicinal Products Act—Civil Protection Exception Ordinance by the Federal Ministry of Health and the European funded Emergency Support Instrument of the European Commission during the observational period [17, 18]. Therefore, acquisition costs were not considered as expenses of healthcare payers. Healthcare providers eligible to administer remdesivir were pre-selected by the Permanent Working Group Competence and Treatment Centres for high consequence infectious diseases (STAKOB) [19]. We further assumed median time to recovery retrieved from the ACTT-1 to be equivalent with LOS.

### Ethical considerations

Due to the retrospective study design and a pseudonymised documentation of underlying cost data, no ethical vote was needed. Moreover, accordign to Health Data Protection Act of North-Rhine Westphalia (GDSG NW), no patient informed consent was necessary for this study [20].

## Results

### Patient characteristics

**Fig. 1:**
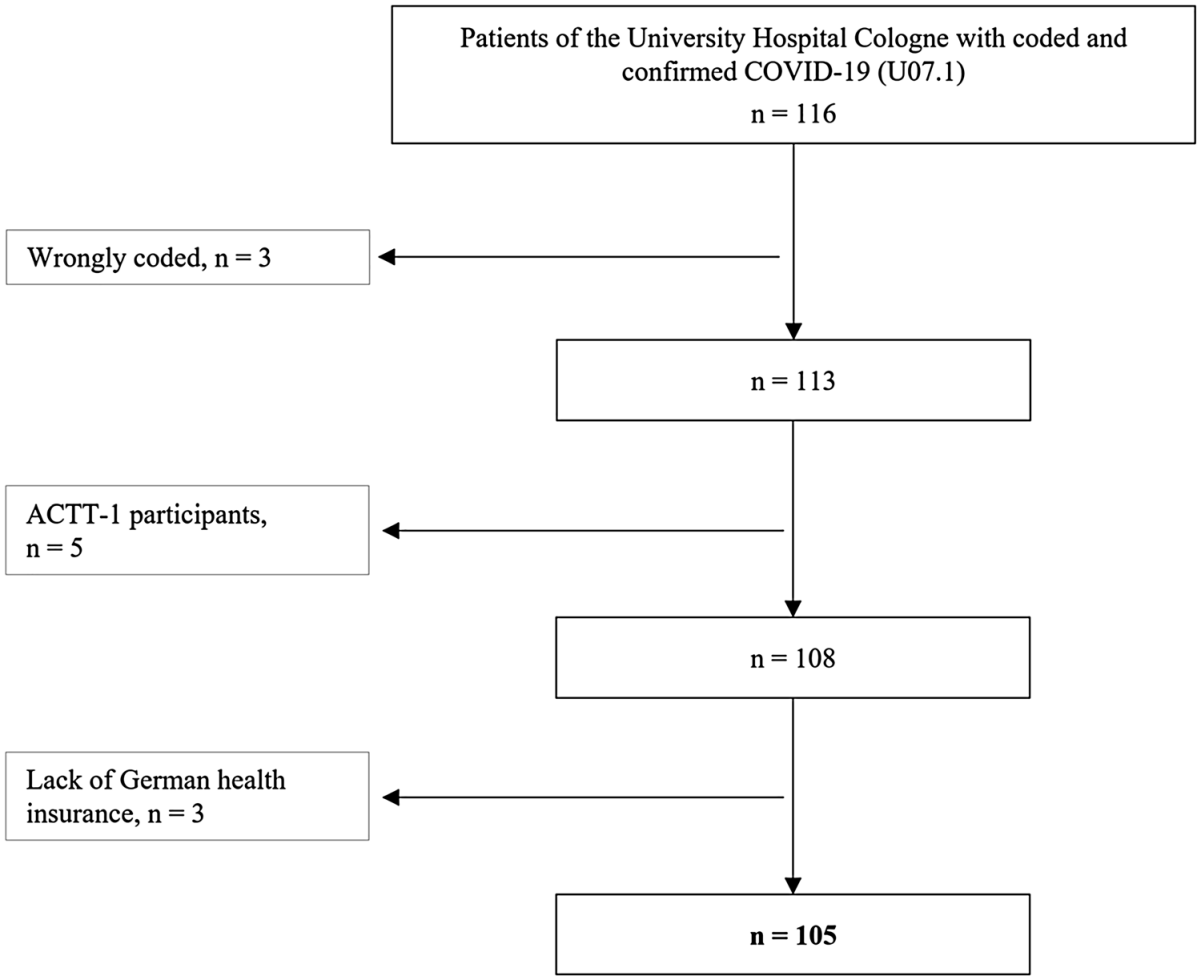
Patient characteristics

116 patients with a confirmed and coded COVID-19 diagnosis were identified for the observational period at initial review of the medical-chart data. Of those, the case-by-case medical-chart review revealed three wrongly coded patients with encoding errors which were therefore excluded from further analyses. According to the pre-defined exclusion criteria, ACTT-1 participants (*n* = 5) and patients with incomplete accounting data due to the lack of German health insurance (*n* = 3) were also excluded. As shown in Fig. 1, the final cohort included 105 patients. Thereof, no patient was hospitalised more than once.

Patient characteristics are shown in Table 1. The median age was 60 years. Nearly 42% of the patients were female. Most patients (81.0%) had a statutory health insurance. The median LOS was 9 days; however, hospitalisation duration ranged from 1 to 168 days. Similarly, the effective CMI, which is a value that demonstrates the case severity and resource consumption per case [21], had a median of 0.74 and ranged from 0.14 to 53.42. Among 43 patients (41.0%) treated at the intensive-care unit (ICU), 30 patients (28.6% of total patient cohort; 69.8% of patients on ICU) received oxygen by invasive ventilation.

**Table 1:** Patient characteristics

Patients	105
Age (in years)	
Median	60
Mean (range)	57 (0–89)
Gender	
Female (%)	44 (41.9%)
Male (%)	61 (58.1%)
Type of health insurance	
Private (%)	20 (19.0%)
Statutory (%)	85 (81.0%)
Length of stay (in days)	
Median	9
Mean (range)	16.4 (1–168)
Treatment at intensive-care unit (%)	43 (41.0%)
Invasive ventilation (%)	30 (28.6%)
Effective case-mix index	
Median	0.74
Mean (range)	3.35 (0.14–53.42)

**Fig. 2:**
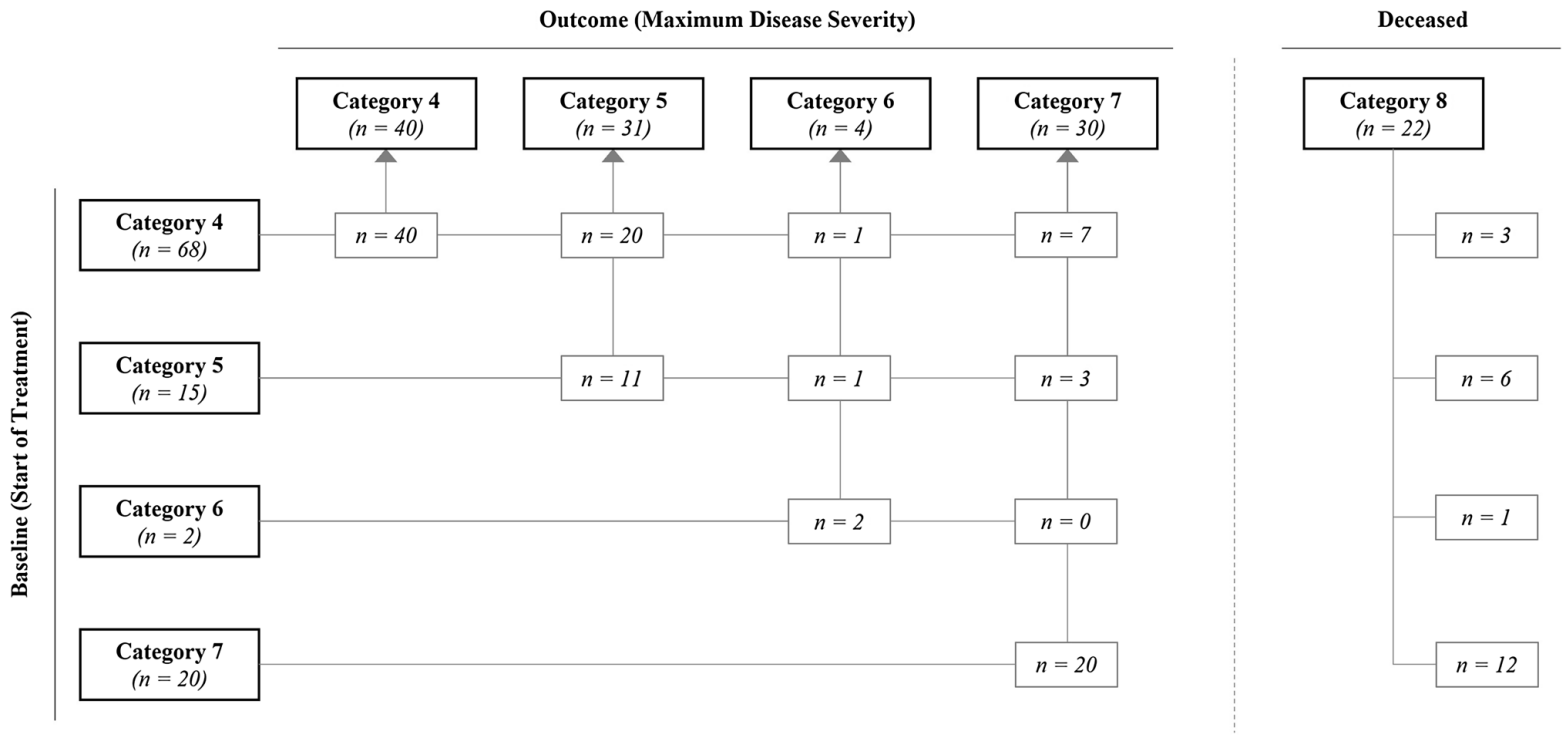
Distribution of COVID-19 patients at baseline and at maximal disease severity grade according to eight-point ordinal scale

As shown in Fig. 2, 68 patients (64.8%) did not receive any type of supplemental oxygen at the time of hospitalisation (category 4). However, in 28 patients (41.2%) with category 4 at baseline, the COVID-19 disease severity increased, and oxygen was supplied in further course. Of category 5 patients at baseline (*n* = 15; 14.3%), four patients (3.8%) worsened to a higher category indicating increasing oxygen need. All patients with category 6 (*n* = 2, 1.9%) or category 7 (*n* = 20; 19.0%) at baseline remained in their categories. For category 7, no switch to a more intense treatment cluster was possible. Deceased patients (category 8: *n* = 22; 21.0%) were analysed according to the patients’ category at the baseline. The mortality rate of the respective baseline categories ranged from 4.4% in category 4 to 60.0% in category 7. About 59% (*n* = 13) of the deceased patients received non-invasive ventilation, high-flow oxygen, invasive mechanical ventilation, or ECMO (categories 6 and 7) at baseline, indicating a severe or critical course of disease.

In categories 4 to 7, 73 patients (69.5%) remained in their baseline categories during the entire hospital stay. The clinical status of 21 patients (20.0%) got worse by one category, while four patients (3.8%) worsened by two categories on the ordinal scale. Seven patients (6.7%) “skipped” two categories and worsened by the maximum of three categories.

### G-DRG and cost analysis

Fifteen different MDC were identified demonstrating a wide range of resource consumption (Table 2). Thereof, 80 patients (76.2%) were grouped to Pre-MDC ‘Care-Intensive/Severe Cases’ (*n* = 25; 23.8%), MDC 04 ‘Diseases and Disorders of the Respiratory System’ (*n* = 46; 43.8%), and MDC 05 ‘Diseases and Disorders of the Circulatory System’ (*n* = 9; 8.6%).

**Table 2:** Performance and G-DRG costs’ analysis

Major diagnosis category (MDC)	G-DRG codes	Patients in *n*	LOS median (range) in *d*	Eff. CMI median (range)	Side diagnoses median (range) in *n*	OPS codes median (range) in *n*	Total treatment costs median (range) in EUR
Pre-MDC: care-intensive/severe cases	A07; A09; A11; A13; A15	25	32 (4–168)	8.69 (1.04–53.42)	7 (1–46)	18 (3–115)	53,070.94 (6620.20–348,861.16)
01-Diseases and disorders of the nervous system	B66; B77; B80	3	8 (2–23)	0.65 (0.28–1.39)	3 (1–11)	9 (1–11)	3503.32 (1428.90–9727.33)
02-Diseases and disorders of the eye	C01; C63	2	3 (1–5)	0.51 (0.20–0.81)	2.5 (1–4)	3.5 (2–5)	2318.89 (938.69–3699.09)
03-Diseases and disorders of the ear, nose, mouth and throat	D63	2	2 (2–2)	0.35 (0.35–0.35)	2 (2–2)	1 (1–1)	1627.32 (1627.32–1627.32)
04-Diseases and disorders of the respiratory system	E36; E40; E71; E77; E79	46	8 (1–56)	0.57 (0.17–7.75)	1 (1–22)	3 (1–19)	3746.86 (860.00–62,216.69)
05-Diseases and disorders of the circulatory system	F06; F43; F62; F67; F70; F71; F73; F75	9	4 (1–28)	0.64 (0.17–5.26)	2 (1–12)	3 (2–10)	3030.39 (822.39–29,923.42)
06-Diseases and disorders of the digestive system	G67	1	9	0.51	1	3	3429.67
12-Diseases and disorders of the male reproductive system	M01; M04	2	6.5 (3–10)	1.63 (0.90–2.36)	2 (1–3)	4 (2–6)	6892.74 (3708.98–10,076.50)
13-Diseases and disorders of the female reproductive system	N01	1	2	2.36	1	4	9001.64
14-Pregnancy, childbirth and puerperium	O60; O65	3	1 (1–4)	0.36 (0.17–0.49)	4 (3–5)	1 (1–3)	1427.44 (890.32–2316.48)
15-Newborn and other neonates (perinatal period)	P67	1	3	0.17	4	2	984.77
17-Hematological and solid neoplasms	R11; R60	3	21 (5–58)	2.19 (1.42–6.07)	5 (3–15)	12 (1–19)	10,634.35 (8898.31–84,812.24)
18A-HIV	S01; S63	3	7 (5–15)	0.57 (0.43–0.74)	8 (1–9)	4 (1–8)	6732.83 (5777.61–7355.97)
18B-Infectious and parasitic diseases	T63	3	5 (2–9)	0.34 (0.34–0.43)	8 (2–8)	2 (1–4)	2190.04 (1541.35–2761.93)
23-Factors influencing health status and other contacts with health services	Z65	1	1	0.20	1	2	922.63
Total value		105	1719	351.98	639	1011	1,976,177.83

*Eff. CMI* effective case-mix index, *G-DRGs* German diagnosis-related groups, *HIV* human immunodeficiency virus, *LOS* length of stay, *MDC* major diagnosis category, *OPS* operating and procedure

Comparing all detected MDC, the longest median LOS was identified in Pre-MDC with 32 days (range 4–168 days). Furthermore, both the highest total treatment costs and the highest effective CMI were observed in Pre-MDC with EUR 53,070.94 (range EUR 6620.20–EUR 348,861.16) and 8.69 (range 1.04–53.42), respectively. Also, the median number of side diagnoses (7; range 1–46) and the median number of OPS codes (7; range 3–115) were both higher than in another MDC.

Forty-six patients were categorised in MDC 04 including patients suffering from respiratory disorders in line with respiratory syndrome caused by the COVID-19 disease. Patients with MDC 04 had a median LOS of 8 days (range 1–56) with a median effective CMI of 0.57 (range 0.17–7.75). This led to median total treatment costs of EUR 3746.86 (range EUR 860.00–EUR 62,216.69). For MDC 04, the median number of side diagnoses was 1 (range 1–22), while the median number of OPS codes accounted for 3 (range 1–19).

### Retrospective modelling of remdesivir for COVID-19 inpatient treatment costs

According to the remdesivir label, patients of category 5 (*n* = 15, 14.3%) and category 6 (*n* = 2, 1.9%) at the time of hospitalisation (baseline) were included in our hypothetical model. Our retrospective analysis was based on median time to recovery as shown in the ACTT-1, indicating for remdesivir a reduced LOS of 2 days for patients of category 5 (7 vs. 9 days in remdesivir vs. placebo) and 4.5 days for patients of category 6 (15 vs. 19.5 days in remdesivir vs. placebo) [14]. For plausibility, patients of baseline category 5 with an LOS ≤ 3 days (*n* = 3, 2.6%) were excluded from further calculations, resulting in a final patient cohort of 14 patients (category 5: *n* = 12 and category 6: *n* = 2).

For both patient clusters (categories 5 and 6), LOS findings from ACTT-1 and total G-DRG costs were identified in the first part of this study. Based on this, the retrospective model theoretically calculated G-DRG costs per day. Considering the reduced LOS when administering remdesivir and assuming a linear resource consumption during the hospital stay, we retrospectively modelled the costs use for both categories 5 and 6 (Table 3). Thereby, an average cost reduction for both categories of EUR 2087.25 per patient [excluding drug costs; standard deviation (SD): EUR 1510.20] was determined.

**Table 3:** Retrospective cost assessment of remdesivir inpatient treatment

	G-DRG code	LOS in days	Treatment costs in EUR	Treatment costs per day in EUR	Cost savings due to administration of remdesivir in EUR
Baseline category 5	A09B	45	86,168.83	1914.86	3829.73
	A09C	34	76,937.45	2262.87	4525.73
	A13E	26	32,025.44	1231.75	2463.50
	E77D	4	3010.95	752.74	1505.48
	E79B	9	4597.02	510.78	1021.56
	E79B	23	11,135.49	484.15	968.30
	E79B	22	10,121.24	460.06	920.11
	E79C	4	2060.92	515.23	1030.46
	E79C	7	3000.51	428.64	857.29
	E79C	7	3501.22	500.17	1000.35
	E79C	8	3300.39	412.55	825.10
	S63B	7	5777.61	825.37	1650.75
Baseline category 6	E77C	14	11,273.60	805.26	3623.66
	E36Z	56	62,216.69	1111.01	4999.56
	Mean (SD)	19 (± 16.34)	22,509.10 (± 29,873.15)	872.53 (± 578.68)	2087.25 (± 1510.20)

*G-DRGs* German diagnosis-related groups, *LOS* length of stay, *SD* standard deviation

**Fig. 3:**
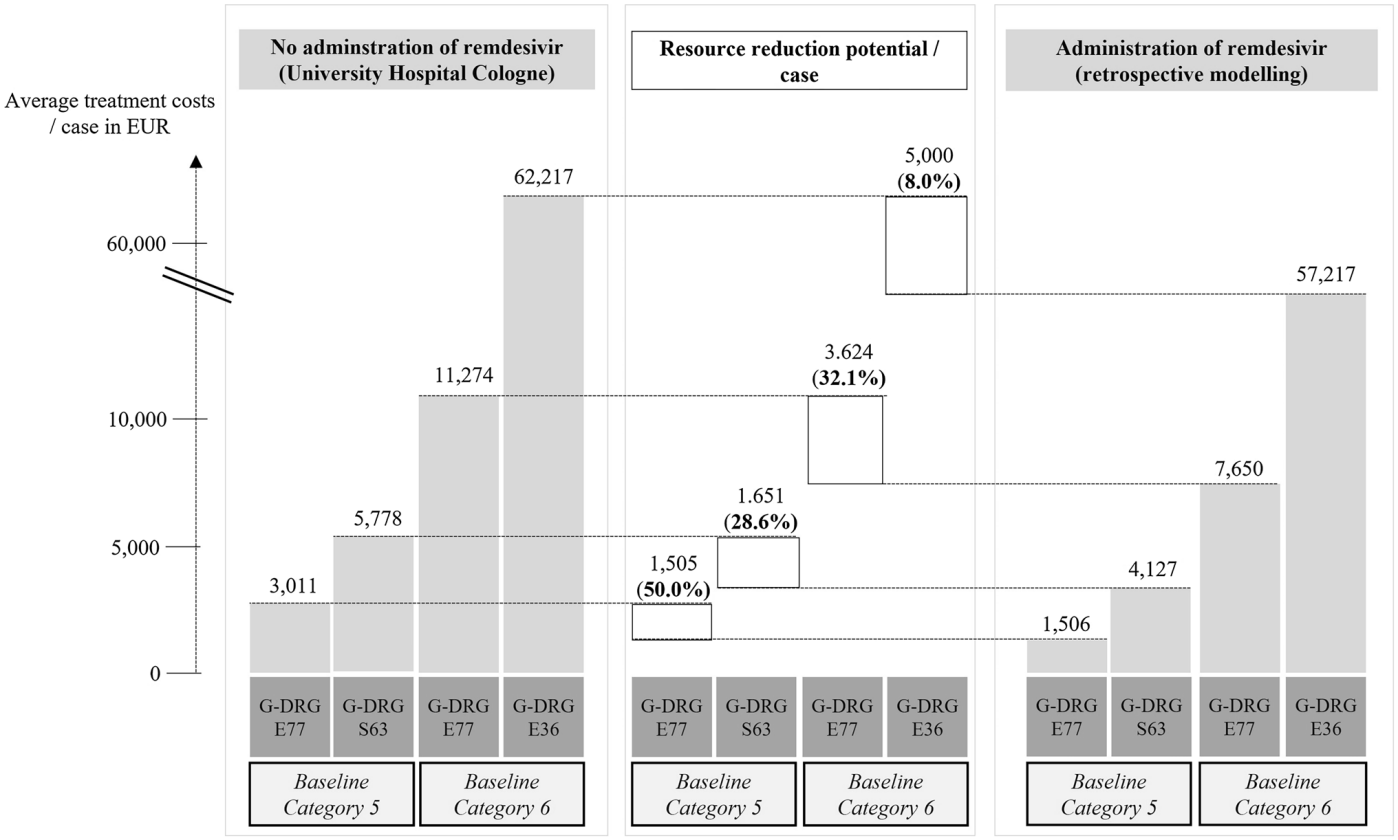
Cost saving potential in exemplary G-DRG codes

As shown in Fig. 3, cost reduction per patient was evaluated by comparing treatment costs retrieved from real-life COVID-19 inpatients with retrospectively modelled costs under consideration of administering remdesivir. We illustratively included G-DRG codes E77/S63 representing baseline category 5 and E77/E36 for baseline category 6. Thus, reduction of treatment costs ranged from 8.0% (E36 in category 6) to 50.0% (E77 in category 5) of the total averaged costs.

## Discussion

We evaluated performed medical services and reimbursement-related indicators of patients with different severities of their COVID-19 disease. Furthermore, we theoretically assessed the impact of administering remdesivir on treatment costs by retrospectively modelling inpatient resource use.

Among the patient cohort, we found a wide range of both main diagnoses and G-DRG codes which were triggered by various causes of different organ entities. In line with the current state of knowledge, it has been shown that COVID-19 manifests not only in lungs, but multiple organ systems leading to systemic hyperinflammation and to different disease courses and severities, accordingly [22–24]. Thus, a wide range of costs per patient from EUR 922.63 to EUR 53,070.94 (median) was observed. We found that 73 patients (69.5%) remained in their clinical baseline category, 40 of whom (54.8%) did not receive any supplemental oxygen during their hospital stay. Yet, the clinical status of 32 patients (30.5%) worsened to more severe stages, indicating the heterogeneous courses of COVID-19. Comparing our results with information provided by the Robert-Koch Institute (RKI-German federal institute under the administration of the German Federal Ministry of Health), however, we found notable deviations. While we analysed 43 ICU patients (41.0%) of which 30 patients (69.8%) received invasive ventilation, the RKI observed a relative distribution of 14% (*n* = 3418) and 23% for both conditions, respectively [25]. Based on the retrospective model, we showed an average cost reduction of EUR 2087.25 per remdesivir eligible patient due to remdesivir. As these cost savings are based on the reduced hospitalisation time (by 2 and 4.5 days, in category 5 and 6 respectively), earlier hospital discharge could lead to a significant reduction of hospital´s occupancy rates during the COVID-19 pandemic. Considering remdesivir costs (5-day treatment course), the model indicated that the cost ratio between medication and treatment costs in Germany is almost balanced (remdesivir acquisition EUR 2070.00 [26, 27] vs. EUR 2087.25 LOS cost savings). As shown in the ACTT-1, targeted and early treatment with remdesivir may not only positively influence LOS but also may prevent costly progression rates to more severe states of disease (i.e., transfer to the ICU) for patients suffering COVID-19 [14]. However, this effect was not further assessed within this study. Resource savings and capacity protection for nursing personnel, physicians, and medical infrastructure seem to be likely but demand further research. Moreover, the balance between medication and treatment costs in the international context may differ compared to this study due to country-specific regulations regarding the acquisition of remdesivir—for instance, remdesivir may be not or not fully reimbursed in international healthcare systems outside the European Union. It is therefore necessary to verify this study’s results applicable for the German healthcare system for other ones. Additionally, the aforementioned balance of medication and treatment costs may further be no longer current after the free-of-charge provision of remdesivir in Germany is expired. If not reimbursed through an additional fee of novel methods of diagnosis and treatment (NUB), remdesivir would technically be included in the G-DRG tariffs which probably do not cover the medication costs of a 5-day or 10-day treatment.

### Methodological considerations

Although best practices for cost analysis were applied, this study has some limitations. The retrospective modelling of this study was mainly based on findings shown in the ACTT-1 [14]. Further literature such as the SOLIDARITY study by the World Health Organisation or the current living guideline by the European Respiratory Society (ERS) does not recommend the use of remdesivir to COVID-19 inpatients who do not require invasive mechanical ventilation [6, 28]. The latter further conditionally recommends the use of remdesivir to COVID-19 inpatients who require invasive mechanical ventilation which implies a preceding assessment of patient-individual risks and benefits that may result from the administration of remdesivir. Both recommendations were based on moderate quality of evidence [28]. Yet, the study at hand attempted to provide first possible economic effects of the administration of remdesivir in COVID-19 inpatients for healthcare payers. To make the retrospective modelling approach as accurate as possible, at this time, best-available data in form of actual G-DRG tariffs retrieved from real-life COVID-19 inpatients were taken as a basis. Nevertheless, additional large clinical trials are urgently needed: first, to further investigate clinical endpoints to confirm findings of ACTT-1 and second, to collect and analyse direct medical costs of the study population to cover health-economic effects [14, 28].

As the data basis of this study is extracted from the medical controlling system, the definite data validity is dependent on certain external influences, i.e., the reimbursement catalogues. As treatment invoices of four patients (3.8% of the total cohort) were under recourse up to the time of evaluation, the final total treatment costs for these patients were lacking. However, we have included the preliminary cost data based on the determined effective CMI. Thus, we were able to include cost data of every patient in the analyses. Moreover, as the analysis is based on medical-chart data of the University Hospital Cologne, the underlying case severity (given by the CMI) might be higher compared to other German non-academic hospitals. However, the high level of bundled expertise within a tertiary care hospital allows the treatment of all COVID-19 patients, irrespective of case severity. Therefore, the data provide insights into as many courses of the COVID-19 disease in real life as possible.

The calculation of cost savings based on the retrospective resource modelling of remdesivir is explicitly related to the reduction of LOS. Reduction of LOS thereby reflected a surrogate to apply median time to recovery defined in the ACTT-1 as “either discharge from the hospital or hospitalisation for infection-control purposes only” to hospital controlling data retrieved from the real-life setting [14]. Moreover, lower and upper thresholds of the respective G-DRG codes were not considered in the calculations for two reasons: first, the calculation has solely modelled potential cost savings of remdesivir. Actual cost savings may vary due to differences in patient characteristics and in G-DRG codes, based on the underlying service-relevant parameters. Second, the simplicity of the model comes along with a high degree of comprehensibility, aiming at making findings transferable into broad clinical practice.

## Conclusion

The main goal of this evaluation was to detect the economic burden from a healthcare payer perspective for inpatient COVID-19 treatment during the first pandemic wave in Germany. To our knowledge, this is the first cost of illness study considering treatment costs of real-life COVID-19 inpatients in Germany. Our findings showed that the collective of COVID-19 inpatients is characterised by a broad spectrum of both G-DRG codes and courses of disease. Findings of the retrospective model further carefully allow to conclude that innovative COVID-19 medications offer effective treatment options for patients. Thereby, hospital financial resources could be saved which therewith could represent an integral part of a comprehensive pandemic control strategy.

The integration of innovative medication, such as remdesivir, into clinical practice showed potential to support disburdening the healthcare system. However, novel reimbursement structures enabling efficient and accelerated access to such drugs are needed for promising innovations. This would majorly contribute to a rapid transfer and integration of innovation into clinical routine care and to a reduction in the system’s burden.

Furthermore, an optimised resource allocation leading to a greater level of efficiency would prevent a healthcare system damage during a pandemic. Thereby, the focus should be on the planning and coordination of the two most relevant resources: experienced medical staff and hospital capacities. Reducing LOS, remdesivir counteracts the high utilisation of both. These topics are closely interrelated and rely on both current infection rates and medium-term planning skills. Therefore, we encourage debating further approaches that may optimise resource use in times of scarce resources and stressed healthcare systems. We suggest combining different measures for future research including the health-economic data to disburden the healthcare system to fight the spread of COVID-19. Innovative treatment strategies, vaccination, infection-control rules, and social distancing are indispensable to control the pandemic.

## Data Availability

Patient-individual cost data will not be made available to others.

## Footnotes

1 Systematically developed, published by a workgroup consisting of representatives of multiple German expert associations (AWMF).

## References

[1] Cheng ZJ, Shan J (2020). 2019 Novel coronavirus: where we are and what we know. Infection.

[2] Böhmer MM, Buchholz U, Corman VM, Hoch M, Katz K, Marosevic DV (2020). Investigation of a COVID-19 outbreak in Germany resulting from a single travel-associated primary case: a case series. Lancet Infect Dis.

[3] European Medicines Agency: Annex I—Summary of Product Characteristics (Veklury). 2020. https://www.ema.europa.eu/en/documents/other/veklury-product-information-approved-chmp-25-june-2020-pending-endorsement-european-commission_en.pdf. Accessed 24 Mar 2021.

[4] Kluge S, Janssens U, Welte T, Weber-Carstens S, Schälte G, Spinner C, et al. S3-Leitlinie - Empfehlungen zur stationären Therapie von Patienten mit COVID-19. AWMF-Register-Nr 113/001. AWMF2021. 10.1055/a-1334-1925 33450783

[5] Siemieniuk R, Rochwerg B, Agoritsas T, Lamontagne F, Leo Y-S, Macdonald H (2020). A living WHO guideline on drugs for covid-19. BMJ.

[6] Pan H, Peto R, Henao-Restrepo AM, Preziosi MP, Sathiyamoorthy V, Abdool Karim Q (2021). Repurposed antiviral drugs for Covid-19—interim WHO solidarity trial results. N Engl J Med.

[7] Li C, Romagnani P, von Brunn A, Anders HJ (2020). SARS-CoV-2 and Europe: timing of containment measures for outbreak control. Infection.

[8] Bundesrechnungshof: Bericht an den Haushaltsausschuss des Deutschen Bundestages nach § 88 Absatz 2 BHO über die Prüfung ausgewählter coronabedingter Ausgabepositionen des Einzelplans 15 und des Gesundheitsfonds. 2021. file:///C:/Users/49160/AppData/Local/Temp/IX1–2021–0572%20-%202021.06.09%20-%20Bericht%20(004).pdf. Accessed 23 July 2021.

[9] § 21 (3) Gesetz zum Ausgleich COVID-19 bedingter finanzieller Belastungen der Krankenhäuser und weiterer Gesundheitseinrichtungen (COVID-19-Krankenhausentlastungsgesetz). Bundesgesetzblatt Jahrgang 2020 Teil I Nr. 142020.

[10] § 21 (5) Gesetz zum Ausgleich COVID-19 bedingter finanzieller Belastungen der Krankenhäuser und weiterer Gesundheitseinrichtungen (COVID-19-Krankenhausentlastungsgesetz). Bundesgesetzblatt Jahrgang 2020 Teil I Nr. 142020.

[11] German Institute for Quality and Efficiency in Health Care: Working Paper Cost Estimation. 2009. https://tools.ispor.org/PEguidelines/source/Germany_WorkPaperCostEst.pdf. Accessed 21 July 2021

[12] Garrison LP, Pauly MV, Willke RJ, Neumann PJ (2018). An overview of value, perspective, and decision context-a health economics approach: an ISPOR special task force report [2]. Value Health.

[13] Internationale statistische Klassifikation der Krankheiten und verwandter Gesundheitsprobleme (10. Revision); German Modification—Version 2021. 2021. https://www.dimdi.de/static/de/klassifikationen/icd/icd-10-gm/kode-suche/htmlgm2021/. Accessed 24 Mar 2021.

[14] Beigel JH, Tomashek KM, Dodd LE, Mehta AK, Zingman BS, Kalil AC (2020). Remdesivir for the treatment of Covid-19—final report. N Engl J Med.

[15] German Institute for the Hospital Remuneration System: Kalkulation von Behandlungskosten – Handbuch zur Anwendung in Krankenhäusern; Version 4.0. 2016. file:///C:/Users/49160/AppData/Local/Temp/Kalkulationshandbuch_4.0_20161010.pdf. Accessed 04 Aug 2021.

[16] Übersicht über die für 2020 gültigen Landesbasisfallwerte in den einzelnen Bundesländern. 2020. https://www.gkv-spitzenverband.de/media/dokumente/krankenversicherung_1/krankenhaeuser/budgetverhandlungen/landesbasisfallwerte/KH_LBFW_2020_2020_04_14.pdf. Accessed 24 Mar 2021.

[17] European Commission: Emergency Support Instrument. https://ec.europa.eu/info/live-work-travel-eu/coronavirus-response/emergency-support-instrument_en. Accessed 09 July 2021.

[18] § 2 Verordnung über die Zulassung von Ausnahmen von Vorschriften des Arzneimittelgesetzes für die Bereiche des Zivil- und Katastrophenschutzes, der Bundeswehr, der Bundespolizei sowie der Bereitschaftspolizeien der Länder; In: AMG-Zivilschutzausnahmeverordnung - AMGZSAV.

[19] Feldt T, Guggemos W, Heim K, Lübbert C, Mikolajewska A, Niebank M, et al. Hinweise zu Erkennung, Diagnostik und Therapie von Patienten mit COVID-19 (Stand 28.04.2021). Ständiger Arbeitskreis der Kompetenz- und Behandlungszentren für Krankheiten durch hochpathogene Erreger am Robert Koch-Institut. 2021.

[20] §6 Gesetz zum Schutz personenbezogener Daten im Gesundheitswesen - Datenverarbeitung für wissenschaftliche Zwecke; In: Gesundheitsdatenschutzgesetz (GSDG NW).

[21] Wozu wird mit dem OPS kodiert? https://www.dimdi.de/dynamic/de/klassifikationen/ops/anwendung/zweck/. Accessed 12 Mar 2021.

[22] Huang C, Huang L, Wang Y, Li X, Ren L, Gu X (2021). 6-month consequences of COVID-19 in patients discharged from hospital: a cohort study. Lancet..

[23] Zhou F, Yu T, Du R, Fan G, Liu Y, Liu Z (2020). Clinical course and risk factors for mortality of adult inpatients with COVID-19 in Wuhan, China: a retrospective cohort study. Lancet.

[24] Durante-Mangoni E, Andini R, Bertolino L, Mele F, Florio LL, Murino P (2020). Early experience with remdesivir in SARS-CoV-2 pneumonia. Infection.

[25] Schilling J, Lehfeld A-S, Schumacher D, Diercke M, Buda S, Haas W, et al. Krankheitsschwere der ersten COVID-19-Welle in Deutschland basierend auf den Meldungen gemäß Infektionsschutzgesetz. J Health Monit. 2020;5.

[26] O’Day D. An open letter from Daniel O’Day, Chairman & CEO, Gilead Sciences. 2020. https://www.gilead.com/news-and-press/press-room/press-releases/2020/6/an-open-letter-from-daniel-oday-chairman--ceo-gilead-sciences. Accessed 16 Mar 2021.

[27] Goldman JD, Lye DCB, Hui DS, Marks KM, Bruno R, Montejano R (2020). Remdesivir for 5 or 10 days in patients with severe Covid-19. N Engl J Med.

[28] Chalmers JD, Crichton ML, Goeminne PC, Cao B, Humbert M, Shteinberg M, et al. Management of hospitalised adults with coronavirus disease 2019 (COVID-19): a European Respiratory Society living guideline. Eur Respir J. 2021. 10.1183/13993003.00048-2021. 10.1183/13993003.00048-2021 PMC7947358 33692120

